# Probiotic properties of a phytase producing *Pediococcus acidilactici* strain SMVDUDB2 isolated from traditional fermented cheese product, Kalarei

**DOI:** 10.1038/s41598-020-58676-2

**Published:** 2020-02-05

**Authors:** Deepali Bhagat, Neelu Raina, Amit Kumar, Meenu Katoch, Yugal Khajuria, Parvez Singh Slathia, Preeti Sharma

**Affiliations:** 1 School of Biotechnology, Shri Mata Vaishno Devi University, Katra, Jammu & Kashmir 182320 India; 2 Instrumentation Division, CSIR - Indian Institute of Integrative Medicine, Canal Road, Jammu 180001 India; 3 Microbial Biotechnology Division, CSIR - Indian Institute of Integrative Medicine, Canal Road, Jammu 180001 India; 4 School of Physics, Shri Mata Vaishno Devi University, Katra, Jammu & Kashmir 182320 India

**Keywords:** Industrial microbiology, Applied microbiology

## Abstract

The nutritional challenge faced by the monogastric animals due to the chelation effects of phytic acid, fuel the research on bioprospecting of probiotics for phytase production. *Pediococcus acidilactici* SMVDUDB2 isolated from Kalarei, exhibited extracellular phytase activity of 5.583 U/mL after statistical optimization of fermentation conditions *viz*. peptone (1.27%); temperature (37 °C); pH (6.26) and maltose (1.43%). The phytase enzyme possessed optimum pH and temperature of 5.5 and 37 °C, respectively and was thermostable at 60 °C. The enzyme was purified 6.42 fold with a specific activity of 245.12 U/mg with hydrophobic interaction chromatography. The purified enzyme had *K*_m_ and *V*_max_ values of 0.385 mM and 4.965 μmol/min respectively, with sodium phytate as substrate. The strain depicted more than 80% survival rate at low pH (pH 2.0, 3.0), high bile salt concentration (0.3 and 0.5%), after gastrointestinal transit, highest hydrophobicity affinity with ethyl acetate (33.33 ± 0%), autoaggregation (77.68 ± 0.68%) as well as coaggregation (73.57 ± 0.47%) with *Staphylococcus aureus* (MTCC 3160). The strain exhibited antimicrobial activity against *Bacillus subtilis* (MTCC 121), *Mycobacterium smegmatis* (MTCC 994), *Staphylococcus aureus* (MTCC 3160), *Proteus vulgaris* (MTCC 426), *Escherichia coli* (MTCC 1652) and *Lactobacillus rhamnosus* (MTCC 1408). The amount of exopolysaccharide produced by the strain was 2 g/L. This strain having the capability of phytate degradation and possessing probiotic traits could find application in food and feed sectors.

## Introduction

With increasing health and nutritional issues, the demand of functional foods is expected to reach 96 billion USD by 2020^1^. Probiotics are playing an important role in functional foods as they tend to increase the nutritional value of food besides increasing the organoleptic properties. LAB (Lactic acid bacteria) producing phytase in the gut can have therapeutic and nutritional aspect associated with them. Phytase in human food will increase the availability of essential divalent mineral ions and release of myo-inositols which can serve as therapeutic measure for various diseases like Crohn’s disease, Alzehmier’s disease, and irritable bowel syndrome. Phytase (myo-inositol hexakisphosphatephosphohydrolases) hydrolyses the phosphoric monoester bonds in phytic acid, a major component of phosphorous in plant diets has a strong chelation with divalent minerals such as Ca^2+^, Mg^2+^, Zn^2+^ and Fe^2+^. The beneficial properties like enzymatic activity, susceptibility or resistance to selected antibiotics associated with probiotic strains depend upon the source of isolation of the bacterial strain^2^. So it is very important to have an appropriate beneficial bacterial strain in food products as the variation in beneficial properties of bacterial strains are related to strain specificity^3^. Reports have suggested that *P. acidilactici* inhabit the entire digestive tract of humans due to their ability to survive in extreme range of pH, temperatures, and osmotic pressures. The importance of *P. acidilactici* has increased with some of the strains of this species have been cited as probiotic bacteria^4^. *P. acidilactici* strain FT28 has been reported for *in vitro* probiotic properties, a probiotic strain with higher nutrient digestibility, heamato- biochemical and antioxidant properties when compared to *Lactobacillus acidophilus* NCDC15^5^. There are reports^6,7^ wherein strains have produced bacteriocins against *Bacillus subtilis* and fungi. *P. acidilactici* (KTU05-7) and *Pediococcus pentosaceus* KTU05-8, KTU05-9. KTU05-8 and KTU05-9 have shown highest extracellular phytase activity^7^ although report on production of exopolysaccharides (EPS) from these strains was absent.Thus, searching new strains of LAB with wider applications is an increasing need of the hour with traditional fermented foods of Southeast Asian countries which are less extensively explored, and some of the species from these sources could be of commercial potential^8^. *P. acidilactici* Kp10, an isolate from a traditional dried curd has an ability to be used into food products because of following properties viz. cell surface hydrophobicity, resistance to proteolytic activities, enzymatic and adhesion associated with it^3^. *P. acidilactici* M76 an isolate from Korean traditional rice wine, *Makgeolli*, was reported for production of exopolysaccharide and as antitumor, antioxidant, antiulcer, antibacterial, immune-stimulating, blood glucose-regulating, and UV radiation protective agent^9^. Most of the EPS-producing LAB strains produce heteropolysaccharides and homopolysaccharides composed of glucose, galactose and rhamnose and others containing only either D-fructose or D-glucose^10^. Health benefits associated with some of the β-D-glucans polysaccharides are antithrombotic, antitumor, or immunomodulatory effect. The effect of EPS, a 2-substituted-(1-3)-β-D-glucan production was observed in case of *Pediococcus damnosus* (strain 2.6) with the change in concentration of glucose, temperature, and bacto casamino acids^11^. Strains of *Pediococcus* reported for production of bacteriocins include *P. acidilactici* Kp10^3^ and *P. acidilactici*^12^. *P. acidilactici* with the capacity for the production of antimicrobial peptide and interesting properties such as resistance to heat, cold, pH and proteolytic treatments have given tremendous importance to the strain^13^. *P. acidilactici* B14 strain has shown resistance to vancomycin and the gene is considered to be inherent in *Pediococcus* genus^13^ but in some strains *P. acidilactici* Kp10^3^ the strain was found to be susceptible to vancomycin. Looking into diversified properties of *Pediococcus* genus, more and more properties of *P. acidilactici* can be explored to obtain a strain having high industrial potentials. *P. acidilactici* strain capable of utilizing pentose sugars such as xylose and arabinose, have shown high resistant towards inhibitors from pretreated lignocellulosic biomass, such as furan derivatives, phenolic compounds and weak acids^4^ during bioethanol production from lignocellulosic biomass. In our study *P. acidilactici* SMVDUDB2 is capable of producing phytase, an exogenous enzyme suggesting potentially important mechanism of probiotic functionality and the ability to survive in gastrointestinal tract conditions; its adhesive properties and potential of producing EPS proves its wider industrial potential.

## Results

### Isolation, selection and identification of phytase producing LAB

Twenty potential LAB isolates obtained from Kalarei samples were capable of surviving at 6.5% NaCl concentrations at pH 3.0 in MRS medium. These 20 presumptive probiotic strains were gram positive and catalase negative and almost all had a phytate degrading ability. The one exhibiting highest extracellular phytate degrading ability (5.18 ± 0.09 U/mL) was identified as *P. acidilactici* based on genotypic identification viz.16 S rRNA gene sequencing and designated as *P. acidilactici* SMVDUDB2. The gene sequence has been submitted in NCBI GenBank and the accession number given is MK280750.

### Phytase studies

Phytase production has been earmarked as an essential criterion of probiotics. Qualitative analysis of phytase in case of *P. acidilactici* SMVDUDB2 showed a zone formation of 8 mm which was higher than *L. rhamnosus* MTCC 1408 (5 mm) on phytase screening medium (PSM) after 48 h at 37 °C while with spectrophotometric analysis the enzyme activity was 5.18 ± 0.09 U/mL and for *L. rhamnosus* (MTCC 1408) phytase activity was 3.69 ± 0.12 U/mL.

### Statistical optimization of phytase production

In order to enhance the phytase production from *P. acidilactici* SMVDUDB2 statistical optimization of various physico-chemical factors was done. Plackett Burman Design (PBD) facilitated the screening of important factors contributing positively to the phytase production^14^. Studies have shown an effect of maltose, peptone, inoculum age, inoculum size, initial pH, incubation temperature and fermentation period on phytase production. Variation in response (phytase production) was observed ranging from 4.93-5.37 U/mL (Supplementary Table 1). The analysis of regression coefficient values illustrated that an incubation temperature, an initial pH, maltose and peptone have significant positive effect on phytase production. The significance of the model (Supplementary Table 2) was stated from results of analysis of the variance (ANOVA) based on PBD.

Determination of the optimal levels and the interaction of the selected variables for an enhanced phytase production in case of *P. acidilactici* SMVDUDB2 was studied using Response Surface Methodology (RSM) and Central Composite Design (CCD). The predicted and observed values of 30 experimental runs is shown in Supplementary Table 3 and the result of analysis of variance (ANOVA) based on CCD experiments is depicted in Supplementary Table 4. The phytase production was predicted by the following regression equation:

$$\begin{array}{c}{\rm{Y}}=+5.37-0.34\,({\rm{A}})-0.10({\rm{B}})+0.072({\rm{C}})-0.34({\rm{D}})-0.48({{\rm{A}}}^{2})\\ \,\,-\,0.22({{\rm{B}}}^{2})-0.54({{\rm{C}}}^{2})-0.43({{\rm{D}}}^{2})+5.625{\rm{E}}-003({\rm{AB}})-0.047({\rm{AC}})\\ \,\,+\,0.16({\rm{AD}})-0.047({\rm{BC}})-0.047({\rm{BD}})-0.31({\rm{CD}})\end{array}$$

where *Y* is the predicted response of phytase production and *A*, *B*, *C* and *D* represent peptone, temperature, pH and maltose respectively.

**Figure 1:**
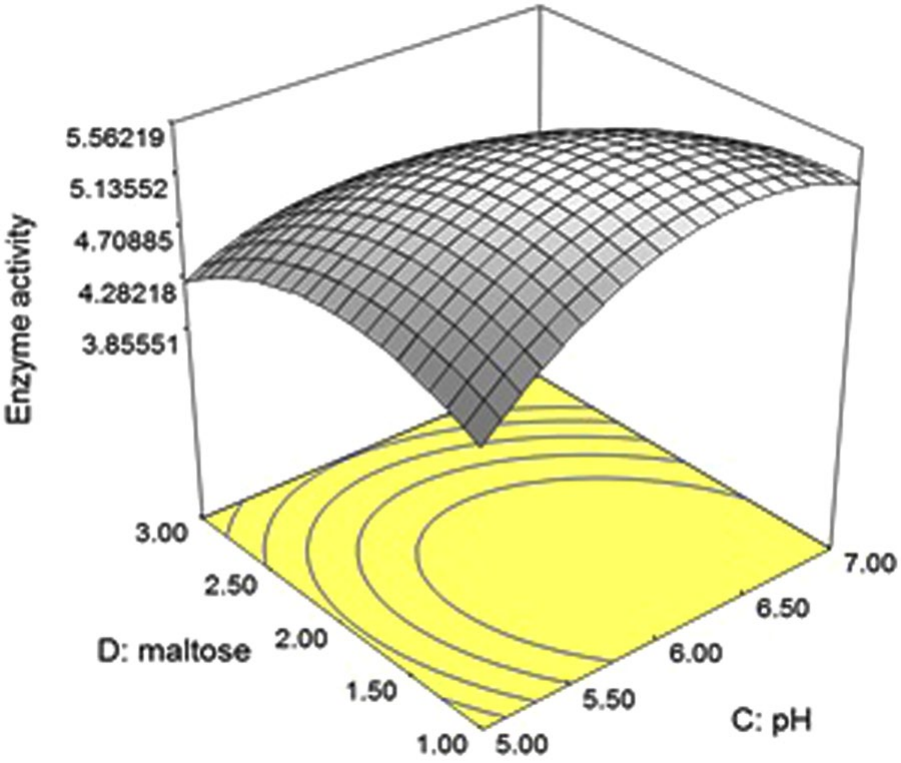
RSM plot showing interactive effect of maltose and pH on phytase production for *P. acidilactici* SMVDUDB2.

Among two positive interacting variables (maltose and pH) an increase in activity was observed with an increase in maltose and pH as shown in the response surface (3D) plot (Fig. 1). The significance of the model was stated by F value 5.27, the probability of the F value is due to noise, the chances for which are only 0.14%. The values of “prob > F” less than 0.05 indicated that in this case A, D, A^2^, C^2^, D^2^, CD model terms are significant. The “Lack of Fit F-value” of 4.61 indicated that a “Lack of Fit F-value” is not significant relative to the pure error. The reliability of the model was stated by coefficient of variation (CV) value 13.87. A high correlation between the predicted and observed response was attributed to the fitness of the model examined by the determination coefficient i.e. R square (0.8310) which is closer to the value of 1.0. In present case the model designated a precision ratio of 6.724, greater than 4 which is the most desirable value indicating an adequate signal response. Point prediction tool of RSM was used for validation of the statistical model. The model was validated by performing the experiment under optimum value of 4 significant variables, peptone (1.27%); temperature (37 °C); pH (6.26) and maltose (1.43%) and the precise closeness of predicted response (5.563 U/mL) and actual response (5.583 U/mL) for phytase production, indicated the suitability of the model. Statistical model has been used to enhance the production of phytase from several microorganisms^15–17^ but, here in we are reporting the optimization of extracellular phytase from *P. acidilactici* using RSM.

### Phytase purification and characterization

Extracellular phytase from *P. acidilactici* SMVDUDB2 was purified using ammonium sulphate precipitation, hydrophobic column chromatography and determined by HPLC analysis as per literature studies^18^. The purification process resulted in 6.42 fold increase in specific activity (245.12 U/mg) with 9.02% yield (Supplementary Table 5). SDS-PAGE and zymogram analysis (Supplementary Fig. 1) confirmed the molecular weight of the protein to be 62 kDa, homogeneity of the purified enzyme has been shown in Supplementary Fig. 2

**Figure 2:**
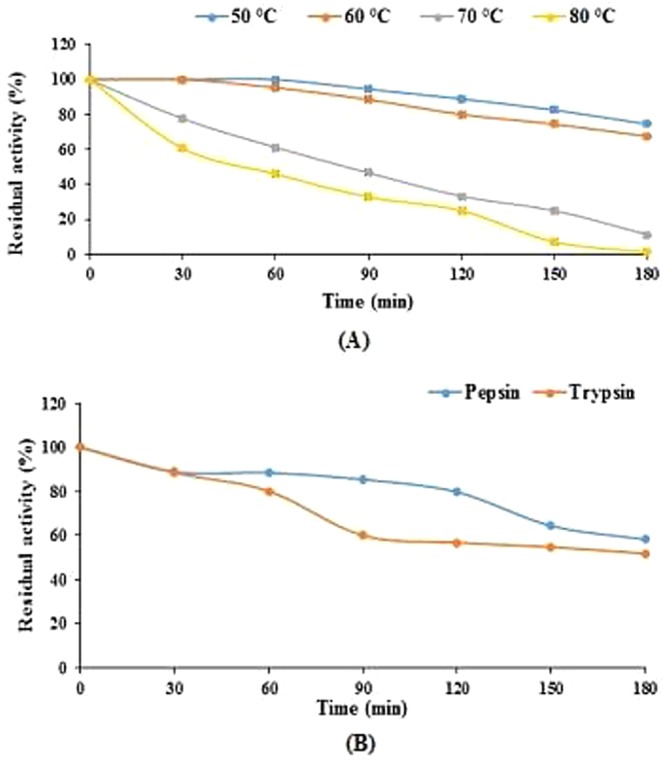
Thermostability and proteolysis resistance of the *P. acidilactici* SMVDUDB2 phytase. (**A**) Thermostability of the purified enzyme. For thermostability, incubation of the enzyme was carried out at 50 and 60 °C for 0–180 min, separately. **(B)** Resistance of purified enzyme towards pepsin and trypsin. Residual phytase activity of the purified phytase was determined after treating with pepsin or trypsin/phytase in the ratio of 1:1 (v/v) for an incubation period of 0–180 min. Data are mean ± SE (n = 3).

The activity of enzyme was maintained over a broad pH range (2.5–12.5) (Supplementary Fig. 3A) and temperature (20–60 °C) (Supplementary Fig. 3B). Enzyme optimum activity was observed at pH 5.5 and temperature 37 °C. The enzyme exhibited good thermostability (Fig. 2A) and the reduction in relative enzyme activity was 25.57% and 32.6% after 3 h of an incubation period at 50 °C and 60 °C respectively, while 38.89% and 54.16% reduction was observed after 1 hour incubation period at 70 °C and 80 °C respectively (Fig. 2A).

The enzyme had strong proteolytic resistance towards pepsin and trypsin at the ratio of 1:1 (pepsin or trypsin/phytase, v/v). Above 80% of phytase activity was retained after 1 hour incubation period in pepsin and trypsin solution, which reduced to 58.21% and 51.81% after 3 h incubation period in pepsin and trypsin solution (Fig. 2B).

The substrate specificity studies for extracellular phytase from *P. acidilactici* SMVDUDB2 revealed that the relative enzyme activity observed was 100% for sodium phytate followed by *p*- nitrophenyl phosphate (80.12 ± 0%), glucose-6-phosphate (79.33 ± 0.79%) and sodium *β*-glycerophosphate (19.99 ± 0%). The maximum enzyme specificity was towards sodium phytate, *K*_m_ and *V*_max_ values were calculated as 0.385 mM and 4.965 μmol/min_,_ respectively (Supplementary Fig. 4) for the same.

## Probiotic Properties

### Ability to withstand simulated gastrointestinal tract (GIT) conditions

**Figure 3:**
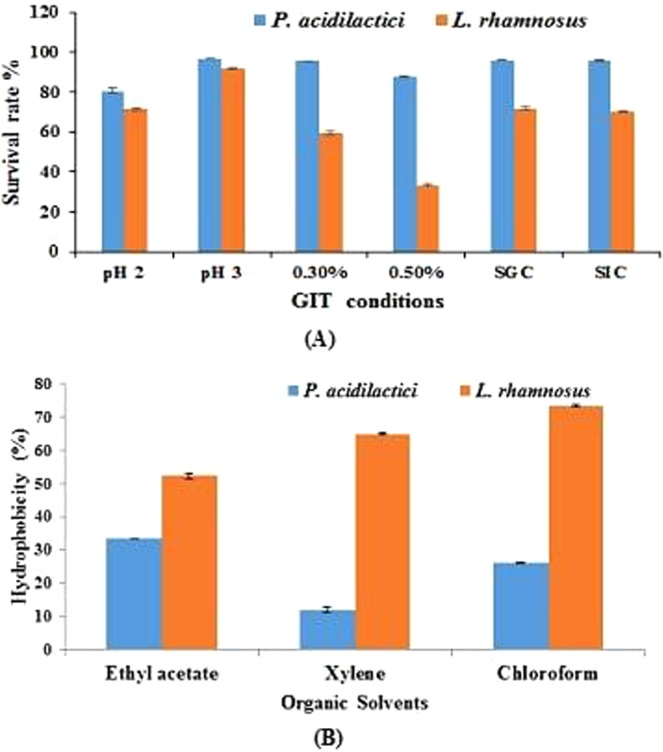
Probiotic properties of *P. acidilactici* SMVDUDB2 **(A)** Survival of *P. acidilactici* SMVDUDB2 and standard probiotic strain *L. rhamnosus* (MTCC 1408) under simulated gastrointestinal conditions. **(B)** Hydrophobicity percent of *P. acidilactici* SMVDUDB2 and standard probiotic strain *L. rhamnosus* (MTCC 1408) with different organic solvents. Data are mean ± SE (n = 3).

*P. acidilactici* SMVDUDB2 showing maximum phytase production was explored for probiotic potentials and its survival rate at high acidic pH (2.0, 3.0), in presence of high bile salts (0.3% and 0.5%), under simulated gastric (pepsin 3 mg/mL, pH 3) and intestinal conditions (trypsin 1 mg/mL, pH 8) was higher as compared standard strain *L. rhamnosus* MTCC 1408 (Fig. 3A). The survival rate above 90% was observed at pH 3.0, 0.3% bile salt concentration and under simulated gastrointestinal conditions in presence of proteolytic enzymes viz. pepsin and trypsin (Fig. 3A). While at pH 2.0 in presence of 0.5% bile salt concentration there was a slight decrease in the survival rate (above 80%).

### Hydrophobicity, autoaggregation and coaggregation studies

In the present study maximum affinity (33.33 ± 0%) was observed towards ethyl acetate (Fig. 3B), an alkalic and monopolar solvent revealing oxidizing (acidic) nature of LAB^3^. The autoaggregation ability was maximum (77.68 ± 0.68%) and a gradual increase is reported after 24 h incubation at 37 °C (Table 1). As per earlier reports *P*. *acidilactici* has shown autoaggregation of 47% after 2 h incubation at 37 °C^19^, but a varying level of coaggregation potential was observed with standard MTCC cultures belonging to gram positive and gram negative pathogenic group, maximum (73.57 ± 0.47%) coaggregation ability was observed with *S. aureus*.

**Table 1:** Study of autoaggregation and coaggregation ability (%) of *P. acidilactici* SMVDUDB2 and standard probiotic bacteria strain *L. rhamnosus* (MTCC 1408) with various bacterial pathogens.

	Autoaggregation %		*B. subtilis*		*M. smegmatis*		Coaggregation %					
							*S. aureus*		*P. vulgaris*			*E. coli*
	4 h	24 h	4 h	24 h	4 h	24 h	4 h	24 h	4 h	24 h	4 h	24 h
*P. acidilactici*	22.76 ± 0.47	77.68 ± 0.68	21.3 ± 0.29	71.18 ± 0.0	18.37 ± 0.19	67.92 ± 0.2	23.32 ± 0.16	73.57 ± 0.47	6.35 ± 0.32	61.67 ± 0.03	16.53 ± 0.25	69.55 ± 0.33
*L. rhamnosus*	26.31 ± 0.34	74.67 ± 0.36	24.29 ± 0.94	58.74 ± 0.2	31.11 ± 0.31	61.64 ± 0.8	38.84 ± 0.23	70.74 ± 0.6	28.68 ± 0.56	79.06 ± 0.4	39.55 ± 0.37	85.93 ± 0.21

Data are mean ± SE (n = 3). Significant difference between 4 h and 24 h (p < 0.05) by the Student’s t test.

*L. rhamnosus* MTCC 1408 exhibited relatively higher (above 50%) hydrophobicity than *P. acidilactici* SMVDUDB2 and maximum affinity was observed with chloroform (73.45 ± 0.47%) while the least affinity was observed with ethyl acetate (52.29 ± 0.67%) indicating the nonacidic and poor electron acceptor property of the strain^19^. *L. rhamnosus* (MTCC 1408) exhibited good autoaggregation ability (74.67 ± 0.36%) after 24 h of incubation at 37 °C and the strain was highly coaggregated with *E. coli* (85.93 ± 0.21%).

### Antibiotic susceptibility

Behaviour of *P. acidilactici* SMVDUDB2 was studied in relation to antibiotics, as elimination of the risk of transmission of antibiotic-resistance genes is an important selection criterion for probiotic bacteria^20^. *P. acidilactici* SMVDUDB2 was found to be sensitive towards cell wall synthesis, (ampicillin, amoxicillin, cefotaxime, cefoperazone, cephalothin, cefepime, imipenem) protein synthesis, (streptomycin, tetracycline, erythromycin, nitrofurantoin, gentamicin, amikacin) RNA synthesis (rifampicin) and DNA synthesis (moxifloxacin) inhibitors. The resistance against norfloxacin, penicillin-G, co-trimoxazole, nalidixic acid, cefpodoxime, ceftazidime and vancomycin was seen. The standard strain *L. rhamnosus* MTCC 1408 was found to be susceptible towards norfloxacin, cefpodoxime, cell wall synthesis, protein synthesis, RNA synthesis and DNA synthesis inhibitors mentioned above, however resistance against cefepime, penicillin-G, co-trimoxazole, nalidixic acid, ceftazidime and vancomycin was observed for this strain also.

### Antagonistic activity

Minimum inhibitory concentration (MIC) was determined by using cell free extract (CFE) to study an inhibitory effect against all the pathogenic genera in case of fraction-A (non-neutralized cell-free supernatant) and Fraction B (pH neutralized supernatant) In our case the inhibitory effect was due to organic acid production and no pediocin-like substance was produced by the reported strain. *P. acidilactici* SMVDUDB2 has shown lactic acid yield (18.32 ± 0.30 mg/mL) comparable with *L. rhamnosus* MTCC 1408 strain (16.99 ± 0.17 mg/mL). MIC value of the potential probiotic LAB strain was 6% to inhibit the growth of *Bacillus subtilis*, *Mycobacterium smegmatis*, *Staphylococcus aureus*, *and Proteus vulgaris* while 8% of MIC was observed against *Escherichia coli*. The importance of organic acids as strong inhibitors against Gram negative bacteria has been reported, whereby the lactic acid acts as permeator of the outer membrane of these bacteria^13,21^.

A higher MIC value was observed i.e. 10% to inhibit *B*. *subtilis* and 12% to inhibit *M. smegmatis*, *S. aureus*, *P. vulgaris* and *E. coli* was observed in case of MTCC 1408.

### Exopolysaccharide (EPS) production

**Figure 4:**
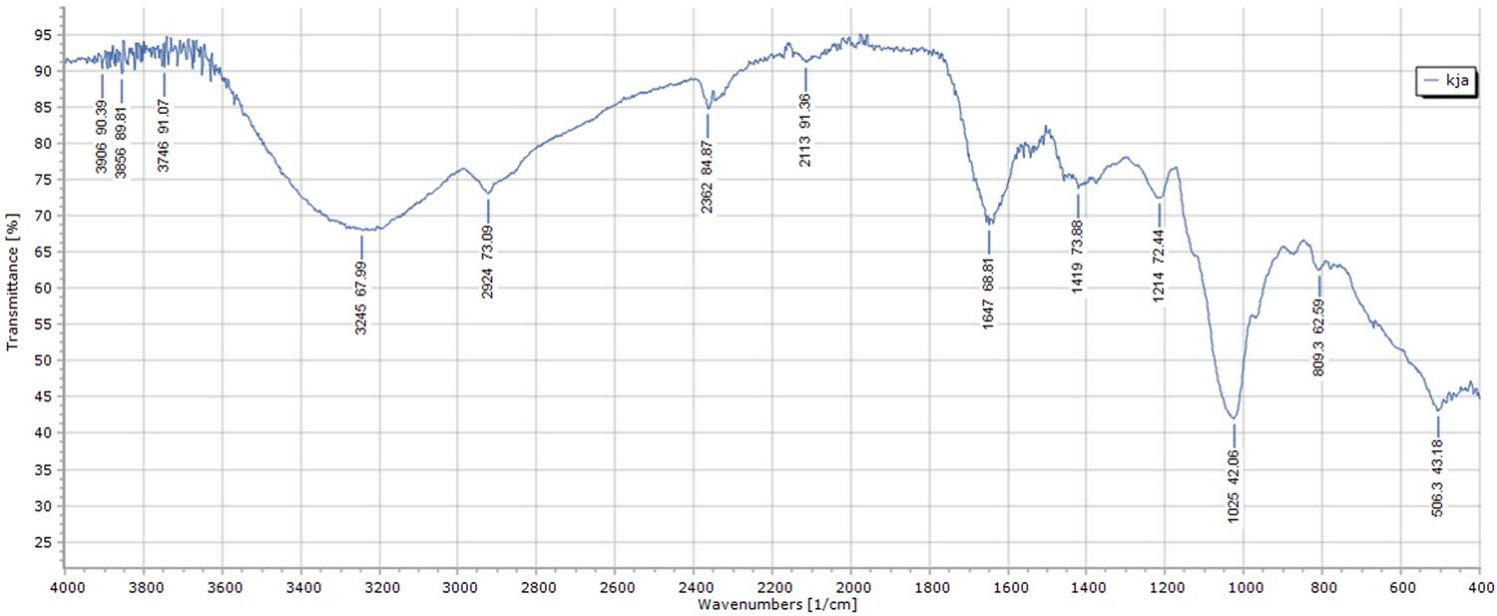
FTIR spectrum of purified EPS from *P. acidilactici* SMVDUDB2 in the range of 400–4000 cm^−1^.

The present strain reported 2 g/L of EPS. The FTIR spectra of EPS from *P. acidilactici* SMVDUDB2 is presented in Fig. 4. A continuous absorption at around 3245 cm^−1^ is assigned to the hydroxyl group (O-H), which is a characteristic of carbohydrate ring. Strong bands around 2924 cm^−1^ corresponded to stretching vibration of the methylene group (C-H), usually present in hexoses^22^. The band at 1647 cm^−1^ was associated to the stretching vibration of carboxyl group (C = O)^23^. Absence of peak around 1529.8 cm^–1^ which corresponds to an amino group was absent in our case. A peak at 1214 cm^−1^ corresponded to the presence of C-O stretching in ether or alcohol groups^24^. The peak around 1025 cm^−1^ recommended the presence of C-O-H link bond position^22^. The spectra exhibited the typical bands of polysaccharides which is within the fingerprint region (1200–950 cm^−1^)^23^. In addition the peak at around 809.3 cm^−1^ was characteristic for mannose^22,25^. Our observation reported the presence of mannose monomer in EPS from *P. acidilactici* SMVDUDB2 and absence of glucuronic acid or diacetyl ester monomeric unit.

## Discussion

The ability of phytase to hydrolyze phytic acid and release phosphate from phytate^26^ has highlighted the lactic acid fermentations of corn, lentils and peas. Lactic acid bacterial strains designated as probiotics isolated from dairy product sources have fulfilled the Food and Drug Administration (FDA) category and recognized as safe. “GRAS” status has been assigned to them and they have been found to be fit for use in fermented dairy and non-dairy products^27^. To apply the effect of probiotics, these microorganisms should be in an adequate amount i.e. 10^6^ colony-forming units (CFU) per gram or milliliter of product when consumed. Their survival in GIT conditions should be such that it provides therapeutic benefits along with phytate degrading ability which is an important criteria of potential probiotic strains^28^. Intrinsic resistance towards vancomycin is a trait reported in probiotic groups, belonging to *Lactobacilli*, *Pediococci* and *Leuconostoc* genera^29,30^. Resistance against vancomycin and cefotaxime in case of *P. acidilactici* B14 strain has been reported and vancomycin resistance gene is inherited in *Pediococcus* genus^13^.

*Pediococcus* is commonly known LAB belonging to homofermentative group capable of catabolizing one mole of glucose in presence of limited oxygen in Embden–Meyerhof–Parnas (EMP) pathway to yield lactic acid only^31^. Reports have shown that production of lactic acid results in lowering of the pH in case of LAB, resulting in lowering or stopping the growth of bacterial pathogens and sometimes even killing them^32^. No pediocin activity was exhibited by *P. acidilactici* SMVDUDB2, but statistical optimization of medium components and fermentation parameters may result in bacteriocin production^33^. According to a recent report intracellular stimulation by internalized inulin nanoparticles lead to enhancement in pediocin production by *P. acidilactici*^12^.

In present study, *P. acidilactici* SMVDUDB2 has fulfilled an essential prerequisite ability of probiotic strain to survive in harsh GIT environment with survival rate higher than the literature reports (*P*. *acidilactici* B14) survival rate was 45.9% at pH 2.5; 72.4% in 0.3% bile salt concentration and 95.8% after passing through gastrointestinal tract viz. pH 4.0^13^.

Our strain has shown diversified properties such as phytase production, EPS producing ability and a survival rate more than 80% at low pH, high bile salt concentrations (0.3% and 0.5%). Its adhesive property is expressed in terms of hydrophobicity. Highest affinity was seen with ethyl acetate whereas lowest with xylene. The lowest affinity (11.85%) to xylene in our case is comparable to *P. acidilactici* (10.4%)^19^. Autoaggregation and coaggregation properties of these probiotic good bacteria make them predominant over the pathogenic bacteria as they outpass the competition with pathogenic coubterparts for the points of attachment on the GIT and because of the competition for nutrients^34^. Adhesion and colonization of intestinal wall by probiotic bacteria is based on the surface properties of the strain^20^. Variations in the properties do exist at strain, species and genus levels and the health benefits are associated with a strain of a particular genus or species which may not be extrapolated to other members of same genus/species. Therefore, each bacterial probiotic strain needs to be investigated. Reports have suggested that coaggregation ability is strain specific against indicator strains and the present study is very much in accordance with earlier reports^35^. Food associated LAB are known as potential producers of EPS^31^ and their EPS can be used as prebiotics^36^. Prebiotics are non-digestible polysaccharides, which act as substrates for fermentation by gut symbionts and stimulate the colonization of beneficial bacteria. However, owing to the lack of expansive knowledge on EPSs from the food-associated LABs, EPSs have remained largely underexploited^9^. Structural analysis of *P. acidilactici* M76 by using HPLC, thin-layer chromatography (TLC) and FTIR has confirmed presence of a glucan consisting of glucose units^9^. On comparison of FTIR graph similar peaks were observed in our study, however the absence of glucuronic acid and diacetyl ester in EPS was ascertained by the missing characteristic absorption peak around the region 1700–1770 cm^−1^ ^24^. *P. pentosaceus* P 773, isolated from a spoiled beer produced 3.9 g/L of EPS in presence of 100 g/L of sucrose for 48 h 37 after 48 h fermentation period^37^.The constituents of EPS were glucose and fructose residues present in ratio of 3:1. *P. acidilactici* M76 produced more than 2.0 g/L of EPS^9^ comparable to yields obtained from *P. acidilactici* SMVDUDB2 indicating the potential of the strain for industrial applications.

The enhanced extracellular phytase (5.583 U/ml) production was the result of two positive interactive variables viz maltose and pH, exhibiting proteolytic resistance against pepsin and trypsin enzymes in the gastrointestinal conditions. The enzyme reported has shown maximum substrate specificity with sodium phytate as in case of *Lactobacillus coryniformis* wherein the values of *K*_m_ and *V*_max_ were 0.32 mM and 4.83 μmol/min respectively^38^. A good thermostability of an enzyme, an important criteria of interest growing in industrial as well as scientific communities towards thermo-acido-philic enzymes^17^ has been fulfilled in case of *P. acidilactici* SMVDUDB2.

In conclusion, *P. acidilactici* SMVDUDB2 isolated from Kalarei exhibited excellent probiotic properties as compared to standard probiotic strain *L. rhamnosus* (MTCC 1408). Phytase producing ability of this probiotic strain is considered as one of the potentially important mechanism of probiotic functionality, its high surviving ability in gastrointestinal tract conditions; its adhesive properties and potential of producing EPS under statistically optimized enzyme conditions proves its wide industrial potential. The proteolytic resistance property of enzyme along with tolerance over wide pH range and temperature contributes to repository of phytase with different origin, properties and has a bright scope for broader application in industries.

## Materials and Methods

### Media, chemicals and bacterial cultures

Microbiological media, chemicals and biochemicals used in present study were purchased from Sigma- Aldrich Chemical Ltd. (St. Louis, MO, USA), HiMedia Pvt. Ltd. (Mumbai, India) and SD Fine Chemicals (Mumbai, India). Antibiotics used for susceptibility test were procured from HiMedia Pvt. Ltd. (Mumbai, India). The microbial strains: *Bacillus subtilis* (MTCC 121), *Mycobacterium smegmatis* (MTCC 994), *Staphylococcus aureus* (MTCC 3160), *Proteus vulgaris* (MTCC 426), *Escherichia coli* (MTCC 1652) and *Lactobacillus rhamnosus* (MTCC 1408) were purchased from Microbial Type Culture Collection (MTCC), Institute of Microbial Technology (IMTECH), Chandigarh, India.

### Isolation of lactic acid bacteria (LAB)

Twelve samples of Kalarei, an indigenous fermented cheese product, were collected from urban and rural areas of Jammu region for isolation of probiotic bacteria. For isolation of LAB serially diluted Kalarei samples were spread on de Man, Rogosa and Sharpe (MRS) agar medium and incubated at 37 °C for 48 h under anaerobic conditions. Presumptive identification was done based on gram staining, microscopic examination and catalase production. The strains were sub-cultured and maintained on MRS agar slants. For long term preservation glycerol stock (50%, v/v) were maintained at −80 °C in MRS.

### Screening of cultures for phytase enzyme

Phytase studies were carried out by growing the bacterial cultures in modified MRS broth (MRS-MOPS) in which inorganic phosphate (KH_2_PO_4_) was replaced by 0.65 g/L of sodium phytate 0.1 M 3-(N-Morpholino) propanesulfonic acid (MOPS). In order to reduce the final phosphate content and to promote phytase production, in the above stated media, the contents of glucose, beef extract and yeast extract were reduced to 10, 4 and 2 g/L, respectively^39^. Modified MRS-MOPS medium was inoculated with 10% (v/v) of 48 h grown cultures (10^8^ CFU/mL) and incubated under anaerobic conditions at 37 °C.

For studying qualitative phytate degrading ability plate assay method was followed^17,40^. The phytase screening media (PSM) was composed of 1.5% glucose, 0.1% sodium phytate, 0.2% NH_4_NO_3_, 0.05% KCl, 0.05% MgSO_4_.7H_2_O, 0.03% MnSO_4_, 0.03% FeSO_4_.7H_2_O and 2.0% agar (pH 7.5). In order to ensure that the zone formation is due to phytate hydrolysis rather than an acid production by the LAB strains, the agar plates were washed using distilled water and petri plates were flooded with 2% (w/v) aqueous cobalt chloride solution. After 5 min of incubation at room temperature the cobalt chloride solution was replaced with a freshly prepared solution containing equal volumes of 6.25% (w/v) aqueous ammonium molybdate) solution and 0.42% (w/v) ammonium meta vanadate solution. After 5 min incubation, the ammonium molybdate/ammonium vanadate solution was removed and the plates were examined for zone of hydrolysis^17^.

For quantitative phytase assay, the amount of liberated inorganic phosphate ions from sodium phytate was measured. The presence of crude extracellular phytase was determined using a reaction mixture consisting of 800 µL sodium phytate (1% w/v) prepared in 0.2 M sodium acetate buffer (pH 5.5) and 200 µL of crude enzyme extract. After an incubation period of 1 h at 37 °C, the reaction was stopped by heating the reaction mixture for 10 min at 100 °C. The liberated phosphate ions were quantified by spectrophotometric method^40^, for this 100 μL of assay mixture was mixed with 900 μL of colour reagent comprised of 1.0 M H_2_SO_4_, 10% ascorbic acid, 2.5% ammonium molybdate (3:1:0.1) (v/v) and after 20 mins of an incubation at 50 °C, absorbance was measured at 820 nm. A standard curve was plotted by using KH_2_PO_4_. One unit of phytase activity was defined as the amount of enzyme that released 1 μmol of phosphate per minute under the assay conditions. Specific phytase activity was defined as U per mg of protein. Protein concentration in the enzyme preparation and during purification steps was determined by Bradford method^41^ using bovine serum albumin as standard. The culture producing highest amount of phytase was selected for further studies.

### Statistical optimization of fermentation conditions for phytase production

Plackett–Burman design (PBD) was employed to determine significant variables for which medium components (carbon and nitrogen sources) and cultivation parameters (initial pH, inoculum age, inoculum size, incubation temperatures and incubation periods) were taken into consideration. Each parameter was examined at two levels (high level and a low level) as illustrated in (Table 2). A total of 12 experimental runs were generated and analysed by using Design Expert 6 software (State-Ease Inc., Minneapolis, U.S.A.).

**Table 2:** Experimental levels of variables for phytase production in Plackett–Burman design.

Symbol Code	Variables	Units	Low level (−1)	High level (+1)
A	Fermentation period	h	43	53
B	Inoculum age	h	43	53
C	Inoculum size	% (v/v)	5	15
D	Incubation temperature	°C	32	42
E	Initial pH	—	5.5	6.5
F	Maltose	% (w/v)	0.5	1.5
G	Peptone	% (w/v)	0.5	1.5
H, J, K, L	Virtual factors	—	−1	+ 1

Central composite design (CCD) of RSM was used for 4 variables (incubation temperature, initial pH, maltose and peptone) identified with significant impact on phytase production. Lower and higher levels of selected variables were depicted in Table 3 and 30 experimental runs were executed. Statistical model was validated based on point prediction method.

**Table 3:** Level of the different independent variables used in CCD for phytase production from *P. acidilactici* SMVDUDB2.

Symbol code	Variables	Unit	Low level	High level
A	Peptone	% (w/v)	1	2
B	Incubation temperature	°C	35	40
C	Initial pH	—	5	7
D	Maltose	% (w/v)	1	3

### Purification and characterization of phytase enzyme

Partial purification of extracellular phytase was carried out using ammonium sulphate precipitation followed by hydrophobic chromatographic studies using Phenyl-Sepharose CL-4B hydrophobic column matrix^42^.

The extracellular enzyme was separated and quantified by a Shimadzu HPLC system (Kyoto, Japan) consisting of an LC-10 ATvp pump, SIL-10 ADvp automatic sampling unit (auto sampler), CTO-10 and SCL-10 Avp as the system controller. Class VP software (version 6.10) was used for data analysis and data processing. The samples were analyzed at 30 °C on RP-18.5 μm, 250 × 4 mm i.d. Merck (Darmstadt, Germany) column. UV detection was performed at 210 nm. The analysis was carried out using a mobile phase comprised of 0.1% trifluroacetic acid in water (solvent A) and acetonitrile (solvent B) at a flow rate of 1.0 mL/min.

The apparent molecular mass of the purified enzyme was estimated by SDS-PAGE according to Laemmli^43^. Electrophoresis was carried out in 12% acrylamide gel using vertical Mini-PROTEAN gel system (Bio-Rad, China). The proteins were stained with Coomassie brilliant blue. The zymogram was prepared by soaking the gel first in 1% Triton X-100 for 1 h at room temperature and then 0.2 M sodium acetate buffer (pH 5.5) at 4 °C for a period of 1 h. Phytase activity was detected by incubating the gel for 16 h in a 0.2 M sodium acetate buffer (pH 5.5) containing 0.4% (w/v) sodium phytate. Activity bands were visualized by immerging the gel in a 2% (w/v) aqueous cobalt chloride solution. After 5 min of incubation at room temperature, the cobalt chloride solution was replaced with a freshly prepared solution containing equal volumes of a 6.25% (w/v) aqueous ammonium molybdate solution and 0.42% (w/v) ammonium vanadate solution. Phytase activity was evident as zones of clearing in an opaque background^44^.

The optimized pH of purified enzyme was determined over the range of 2.5–12.5 using glycine-HCL buffer (pH 2.5), acetate buffer (pH 3.5–5.5), citrate buffer (pH 6.5), glycine–NaOH buffer (pH 8.5–10.5) and KCl-NaOH buffer (pH 12.5).

For optimum temperature studies enzyme substrate complex mixture was incubated at various temperatures viz. 20, 30, 37, 50 and 60 °C. Thermostability test for enzyme was determined by preincubating the purified enzyme at 50 °C, 60 °C, 70 °C and 80 °C for 3 h. The residual activity of thermotolerant enzyme was calculated after a regular interval of 30 min.

Phytase resistance to proteolytic enzymes (pepsin and trypsin) was evaluated according to the method reported in literature^45^ with little modifications. The purified enzyme (10 µg/mL) was incubated in 0.2 M glycine-HCl buffer (pH 2.5) containing pepsin (10 µg/mL) in the ratio of 1:1, and 0.2 M Tris-HCl buffer (pH 8.0) containing trypsin (10 µg/mL) in the ratio of 1:1 at 37 °C for a period of 3 h. As a control, the purified enzyme was incubated in the same condition as stated above but lacking pepsin or trypsin and the percentage residual activity was calculated after a regular interval of 30 min.

Substrate specificity of purified extracellular phytase enzyme was determined using various phosphorylated substrates like sodium phytate, glucose-6-phosphate, sodium *β*-glycerophosphate and *p*- nitrophenyl phosphate at 3 mM concentration. *K*_m_ and *V*_max_ were measured from Lineweaver-Burk plot using different concentrations of sodium phytate (0.1–1.8 mM) prepared in sodium acetate buffer (0.2 M, pH 5.5).

### Molecular identification of selected LAB isolate

Bacterial genomic DNA of potential phytase producing LAB isolate was extracted using DNA purification kit. Identification was based on 16 S rRNA gene sequencing. The universal primers used were (lac1–27 F 5′-AGAGTTTGATCCTGGCTCAG-3′ and lac 1-1492 R 5′ TACGGYTACCTTGTTACGACT-3′). The conditions in PCR programme were as follows: initial denaturation at 94 °C for 2 min, denaturation at 94 °C for 30 s, annealing at 55 °C for 1 min, extension at 72 °C for 1 min and final extension at 72 °C for 10 min. The PCR product obtained was sequenced by AgriGenome Labs Pvt. Ltd., Kerala. Basic Local Alignment Search Tool (BLAST) was used to analyse the homology of sequence and the sequence was submitted in NCBI GenBank. Phylogenetic tree made from sequenced 16 S rRNA region of the isolate and evolutionary analyses were conducted using Clustal Omega.

### Probiotic properties

#### Tolerance to simulated gastrointestinal tract (GIT) conditions

Survival rate was determined at low pH (2.0 and 3.0), high bile salt concentration (0.3% and 0.5%) under simulated GIT conditions as per earlier reports^46,47^ and a comparative study was carried out with standard probiotic bacterial strain MTCC 1408. Evaluation of pathogenicity factor was carried out by studying the lipolytic effect of the isolate^13^.

#### Hydrophobicity, autoaggregation and coaggregation studies

Organic solvents viz. ethyl acetate, xylene, chloroform were used for hydrophobicity studies, autoaggregation and coaggregation^28^ was carried out using MTCC cultures (*B*. *subtilis*, *M*. *smegmatis*, *S*. *aureus*, *P*. *vulgaris* and *E*. *coli*) for evaluating adhesive properties. Similar studies were carried out with standard probiotic strain.

#### Antibiotic susceptibility test

For antibiotic susceptibility test, disc diffusion method was employed as per earlier reports^48^. Petriplates were incubated under anaerobic conditions at 37 °C for 24 h. The plates were examined for zone of inhibition around the antibiotic disks. Twenty two antibiotic discs *viz*. rifampicin (5 mcg), moxifloxacin (5 mcg), ampicillin (10 mcg), amoxicillin (10 mcg), imipenem (10 mcg), gentamicin (10 mcg), norfloxacin (10 mcg), penicillin-G (10 mcg), erythromycin (15 mcg), streptomycin (25 mcg), co-trimoxazole (25 mcg), cefotaxime (30 mcg), cephalothin (30 mcg), cefepime (30 mcg), tetracycline (30 mcg), amikacin (30 mcg), nalidixic acid (30 mcg), cefpodoxime (30 mcg), ceftazidime (30 mcg) and vancomycin (30 mcg)), cefoperazone (75 mcg), nitrofurantoin (300 mcg), were screened against standard probiotic strain MTCC 1408 as well as LAB isolated from Kalarei.

#### Antagonistic activity and organic acid production

Minimum inhibitory concentration (MIC) of cell free extract (CFE) for standard strain MTCC 1408 and LAB isolate was determined^49^ against standard MTCC cultures, *B. subtilis* (MTCC 121), *M. smegmatis* (MTCC 994), *S. aureus* (MTCC 3160), *P. vulgaris* (MTCC 426), *E. coli* (MTCC 1652) grown on nutrient agar medium. For studying antagonistic effect and to determine the presence of bacteriocin like inhibitory substance (BLIS), 48 h grown culture was centrifuged (10,000 × g for 10 min at 4 °C) and the filter sterilized cell free extracts (CFEs) were obtained. Standard MTCC cultures (1% of the bacterial solution having 10^8^ cfu/mL) were inoculated in Luria-Bertani broth (*E. coli*, *S. aureus*) and nutrient broth (*B. subtilis, M. smegmatis and P. vulgaris*) containing CFE of LAB (0, 2, 4, 6, 8, 10 and 12%) and incubated for a period of 24 h at 37 °C under aerobic conditions^49^. The filter sterilized CFEs were assayed as follows: Fraction A, cell-free supernatant and Fraction B, consisted of pH neutralized supernatant (pH 7.0) for detection of inhibition by organic acids^32^. MIP was monitored by measuring optical density (OD600 nm).

Organic acid (lactic acid) production was determined by titration method^50^ for selected LAB isolate as well as MTCC 1408. Amount of organic acid produced from 48 h grown culture in MRS medium at 37 °C was determined by using titration method described by AOAC^50^. Ten millilitre of supernatant from LAB and 3 drops of phenolphthalein as indicator was added for titration against 0.1 M NaOH. The NaOH from the burette was added slowly to the samples until colour changes to pink. Total titratable acidity of lactoc acid (LA) was measured by the following formula:

Total titratable acidity of LA (mg/mL) = mL NaOH × N NaOH × M.E ÷ volume of sample used, where total volume of NaOH used was represented in mL, N denotes normality of NaOH used and mass equivalent (M.E) represents equivalent factor which is equal to 90.08 mg of lactic acid^51^.

#### Exopolysaccharide (EPS) production

The LAB isolate from Kalarei was inoculated in MRS broth supplemented with 1% maltose as carbon source and incubated at 37 °C for 48 h. EPS were isolated from CFE collected after centrifugation at 10000 × g for 30 min at 4 °C followed by ethanol precipitation, dialysis and lyophilization^52^. The concentration of EPS was determined by phenol-sulphuric acid methodology^53^. The EPS sample was analyzed by Fourier- transform infrared (FTIR) spectroscopy. Sample was prepared by mixing lyophilized EPS sample with KBr and the spectra were recorded with a light source in the range of 4000–400 cm^−1^ ^36^.

## Supplementary information

Supplementary Data.
