# Supplementary material for: Probiotic properties of a phytase producing Pediococcus acidilactici strain SMVDUDB2 isolated from traditional fermented cheese product, Kalarei
Source: Sci Rep. 2020 Feb 5;10:1926. doi: 10.1038/s41598-020-58676-2 (PMC7002416; doi:10.1038/s41598-020-58676-2)
Supplement: Supplementary file 1 — Supplementary Data. [file 41598_2020_58676_MOESM1_ESM.docx]

**Supplementary Information**

**Probiotic properties of a phytase producing *Pediococcus acidilactici* strain SMVDUDB2 isolated from traditional fermented cheese product, Kalarei**

Deepali Bhagat^1^, Neelu Raina^1^, Amit Kumar^2^, Meenu Katoch^3^, Yugal Khajuria^4^ Parvez Singh Slathia^1^, Preeti Sharma^1^*

1School of Biotechnology, Shri Mata Vaishno Devi University, Katra, Jammu & Kashmir India-182320

2Instrumentation Division, CSIR - Indian Institute of Integrative Medicine, Canal Road, Jammu-180001

3Microbial Biotechnology Division, CSIR - Indian Institute of Integrative Medicine, Canal Road, Jammu- 180001

4School of Physics, Shri Mata Vaishno Devi University, Katra, Jammu & Kashmir India-182320

*Corresponding author.

Phone: +91 9419304654 (Mob.); E-mail: [preeti.res@gmail.com](mailto:preeti.res@gmail.com)

**Table Captions**

**Supplementary Table 1.** Plackett–Burman-design showing experimental and predicted response for phytase production from *P. acidilactici* SMVDUDB2.

**Supplementary Table 2.** Analysis of variance (ANOVA) results for phytase production from *P. acidilactici* SMVDUDB2 based on Plackett–Burman-designed experiments.

**Supplementary Table 3.** Experimental and predicted response (phytase activity U/mL) for phytase production from *P. acidilactici* SMVDUDB2 using CCD of RSM.

**Supplementary Table 4.** Analysis of variance (ANOVA) results in case of *P. acidilactici* SMVDUDB2 based on CCD experiments (A: Peptone, %; B: Incubation temperature, °C; C: pH; D: maltose, %).

**Supplementary Table 5.** Purification of extracellular phytase from *P. acidilactici* SMVDUDB2.

**Figure Captions**

**Supplementary Figure 1.** SDS-PAGE and zymogram analysis of phytase from *P. acidilactici* SMVDUDB2: Lane 1: protein molecular weight marker, mass (in KDa); Lane 2: crude enzyme preparation; Lane 3: pooled active fractions after ammonium sulphate precipitation; Lane 4: purified enzyme after hydrophobic column chromatography (circled); Lane 5: zymogram of purified phytase showing opaque region in gel (circled).

**Supplementary Figure 2.** HPLC analysis (A) HPLC profile of standard phytase (B) HPLC profile of purified phytase from *P. acidilactici* SMVDUDB2.

**Supplementary Figure 3.** (A) Effect of pH and (B) temperature on phytase activity of *P. acidilactici* SMVDUDB2. Data are mean ± SE (n = 3).

**Supplementary Figure 4.** Lineweaver-Burk plot of phytase from *P. acidilactici* SMVDUDB2 using sodium phytate as substrate.

**Supplementary Table 1.** Plackett–Burman-design showing experimental and predicted response for phytase production from *P. acidilactici* SMVDUDB2.

**Experimental variables* Phytase activity (U/mL)**

| **Run Order** | **A** | **B** | **C** | **D** | **E** | **F** | **G** | **Experimental** | **Predicted** |
| --- | --- | --- | --- | --- | --- | --- | --- | --- | --- |
| 1 | 53 | 45 | 15 | 32 | 5.5 | 0.5 | 1.5 | 5.18 | 5.17 |
| 2 | 43 | 45 | 5 | 32 | 5.5 | 0.5 | 0.5 | 5 | 4.99 |
| 3 | 43 | 53 | 15 | 42 | 5.5 | 1.5 | 1.5 | 5.37 | 5.36 |
| 4 | 53 | 53 | 5 | 42 | 5.5 | 0.5 | 0.5 | 4.93 | 4.94 |
| 5 | 43 | 53 | 15 | 32 | 6.5 | 0.5 | 0.5 | 5 | 5.01 |
| 6 | 53 | 45 | 15 | 42 | 5.5 | 1.5 | 0.5 | 4.93 | 4.94 |
| 7 | 53 | 53 | 15 | 32 | 6.5 | 1.5 | 0.5 | 5 | 4.99 |
| 8 | 53 | 45 | 5 | 32 | 6.5 | 1.5 | 1.5 | 5.37 | 5.38 |
| 9 | 43 | 53 | 5 | 32 | 5.5 | 1.5 | 1.5 | 5.37 | 5.38 |
| 10 | 53 | 53 | 5 | 42 | 6.5 | 0.5 | 1.5 | 5.37 | 5.36 |
| 11 | 43 | 45 | 15 | 42 | 6.5 | 0.5 | 1.5 | 5.37 | 5.38 |
| 12 | 43 | 45 | 5 | 42 | 6.5 | 1.5 | 0.5 | 5.2 | 5.19 |

**Supplementary Table 2.** Analysis of variance (ANOVA) results for phytase production from *P. acidilactici* SMVDUDB2 based on Plackett–Burman-designed experiments. [A: fermentation period (h); B: inoculum age (h); C: inoculum size (% v/v); D: incubation temperature (°C); E: initial pH; F: maltose (% w/v); and G: peptone (% w/v)]

| **Source** | **Sum of Squares** |  | ***df*** | **Mean Square** | ***F* value** | **Prob > *F*** |  |
| --- | --- | --- | --- | --- | --- | --- | --- |
| Model | 0.40 |  | 7 | 0.057 | 120.54 | 0.0002 | significant |
| A | 0.023 |  | 1 | 0.023 | 49.28 | 0.0022 |  |
| B | 8.333E-006 |  | 1 | 8.333E-006 | 0.018 | 0.9010 |  |
| C | 0.013 |  | 1 | 0.013 | 26.68 | 0.0067 |  |
| D | 5.208E-003 |  | 1 | 5.208E-003 | 10.96 | 0.0296 |  |
| E | 0.023 |  | 1 | 0.023 | 49.28 | 0.0022 |  |
| F | 0.013 |  | 1 | 0.013 | 26.68 | 0.0067 |  |
| G | 0.32 |  | 1 | 0.32 | 680.86 | < 0.0001 |  |
| Residual | 1.900E-003 |  | 4 | 4.750E-004 |  |  |  |
| Cor Total | 0.40 |  | 11 |  |  |  |  |

**Supplementary Table 3.** Experimental and predicted response (phytase activity U/mL) for phytase production from *P. acidilactici* SMVDUDB2 using CCD of RSM.

**Experimental Variables Phytase activity (U/mL)**

| **Run** | **A: Peptone %** | **B: Incubation**  **temperature** | **C: pH** | **D: Maltose**  **%** | **Experimental** | **Predicted** |
| --- | --- | --- | --- | --- | --- | --- |
|  |  | **(°C)** |  |  |  |  |
| 1 | 1 | 35 | 7 | 1 | 5.41 | 5.07 |
| 2 | 1 | 40 | 7 | 1 | 5.41 | 4.86 |
| 3 | 1 | 35 | 5 | 1 | 5 | 4.13 |
| 4 | 1.5 | 32.5 | 6 | 2 | 4.16 | 4.67 |
| 5 | 1 | 35 | 5 | 3 | 3.75 | 3.83 |
| 6 | 1.5 | 37.5 | 6 | 2 | 5 | 5.37 |
| 7 | 2 | 35 | 5 | 3 | 3.33 | 3.56 |
| 8 | 2.5 | 37.5 | 6 | 2 | 2.98 | 2.79 |
| 9 | 1.5 | 37.5 | 8 | 2 | 2.91 | 3.37 |
| 10 | 1 | 35 | 7 | 3 | 3.75 | 3.55 |
| 11 | 1.5 | 37.5 | 4 | 2 | 2.91 | 3.08 |
| 12 | 1.5 | 37.5 | 6 | 2 | 5.41 | 5.37 |
| 13 | 0.5 | 37.5 | 6 | 2 | 3.33 | 4.14 |
| 14 | 2 | 40 | 7 | 3 | 2.16 | 2.71 |
| 15 | 1 | 40 | 5 | 3 | 3.75 | 3.62 |
| 16 | 1.5 | 37.5 | 6 | 2 | 4.98 | 5.37 |
| 17 | 1.5 | 42.5 | 6 | 2 | 4.16 | 4.27 |
| 18 | 1.5 | 37.5 | 6 | 2 | 5.62 | 5.37 |
| 19 | 2 | 35 | 7 | 1 | 4.16 | 3.97 |
| 20 | 1.5 | 37.5 | 6 | 2 | 5.62 | 5.37 |
| 21 | 1 | 40 | 5 | 1 | 4.16 | 4.10 |
| 22 | 2 | 40 | 7 | 1 | 4.16 | 3.78 |
| 23 | 1.5 | 37.5 | 6 | 4 | 3.33 | 2.96 |
| 24 | 2 | 40 | 5 | 1 | 3.33 | 3.21 |
| 25 | 1.5 | 37.5 | 6 | 2 | 5.58 | 5.37 |
| 26 | 1.5 | 37.5 | 6 | 0 | 3.33 | 4.33 |
| 27 | 2 | 40 | 5 | 3 | 3.33 | 3.37 |
| 28 | 1 | 40 | 7 | 3 | 3.33 | 3.15 |
| 29 | 2 | 35 | 7 | 3 | 3.33 | 3.09 |
| 30 | 2 | 35 | 5 | 1 | 3.33 | 3.21 |

**Supplementary Table 4.** Analysis of variance (ANOVA) results in case of *P. acidilactici* SMVDUDB2 based on CCD experiments (A: Peptone, %; B: Incubation temperature, °C; C: pH; D: maltose, %).

| **Source** | **Sum squares** | **of** | ***df*** | **Mean square** | ***F* value** | **Prob >F** |  |
| --- | --- | --- | --- | --- | --- | --- | --- |
| **Model** | 23.10 |  | 14 | 1.65 | 5.27 | 0.0014 | Significant |
| A | 2.75 |  | 1 | 2.75 | 8.8 | 0.0096 |  |
| B | 0.25 |  | 1 | 0.25 | 0.79 | 0.3894 |  |
| C | 0.12 |  | 1 | 0.12 | 0.4 | 0.5375 |  |
| D | 2.82 |  | 1 | 2.82 | 9.01 | 0.0089 |  |
| A2 | 6.2 |  | 1 | 6.2 | 19.81 | 0.0005 |  |
| B2 | 1.38 |  | 1 | 1.38 | 4.41 | 0.0532 |  |
| C2 | 7.9 |  | 1 | 7.9 | 25.24 | 0.0002 |  |
| D2 | 5.11 |  | 1 | 5.11 | 16.33 | 0.0011 |  |
| AB | 5.06E-04 |  | 1 | 5.06E-04 | 1.62E-03 | 0.9685 |  |
| AC | 0.035 |  | 1 | 0.035 | 0.11 | 0.7422 |  |
| AD | 0.41 |  | 1 | 0.41 | 1.32 | 0.2689 |  |
| BC | 0.035 |  | 1 | 0.035 | 0.11 | 0.7422 |  |
| BD | 0.035 |  | 1 | 0.035 | 0.11 | 0.7422 |  |
| CD | 1.51 |  | 1 | 1.51 | 4.81 | 0.0444 |  |
| Residual | 4.70 |  | 15 | 0.31 |  |  |  |
| Lack of fit | 4.24 |  | 10 | 0.42 | 4.61 | 0.0528 | Not significant |
| Pure error | 0.46 |  | 5 | 0.092 |  |  |  |
| Cor Total | 27.79 |  | 29 |  |  |  |  |

**Supplementary Table 5.** Purification of extracellular phytase from *P. acidilactici* SMVDUDB2.

| **Steps of purification** | **Total activity (U)** | **Total protein (mg)** | **Specific activity (U/mg)** | **Purification fold** | **Yield (%)** |
| --- | --- | --- | --- | --- | --- |
| Crude enzyme | 5541 | 145 | 38.21 | 1 | 100 |
| Ammonium sulphate precipitation (60-90%) | 1000.5 | 10.5 | 95.29 | 2.49 | 18.06 |
| Hydrophobic interaction Chromatography | 500.04 | 2.04 | 245.12 | 6.42 | 9.02 |


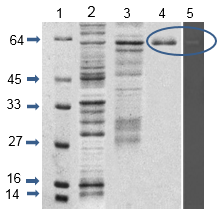


**Supplementary Figure 1.** SDS-PAGE and zymogram analysis of phytase from *P. acidilactici* SMVDUDB2: Lane 1: protein molecular weight marker, mass (in KDa); Lane 2: crude enzyme preparation; Lane 3: pooled active fractions after ammonium sulphate precipitation; Lane 4: purified enzyme after hydrophobic column chromatography (circled); Lane 5: zymogram of purified phytase showing opaque region in gel (circled).


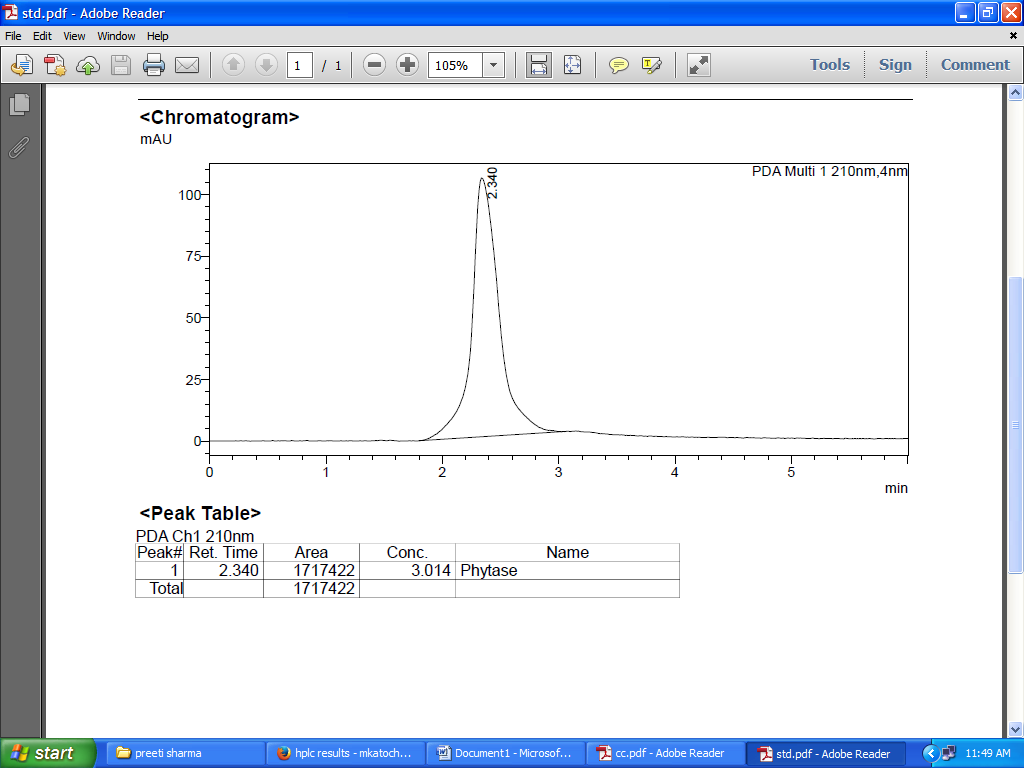


(A)


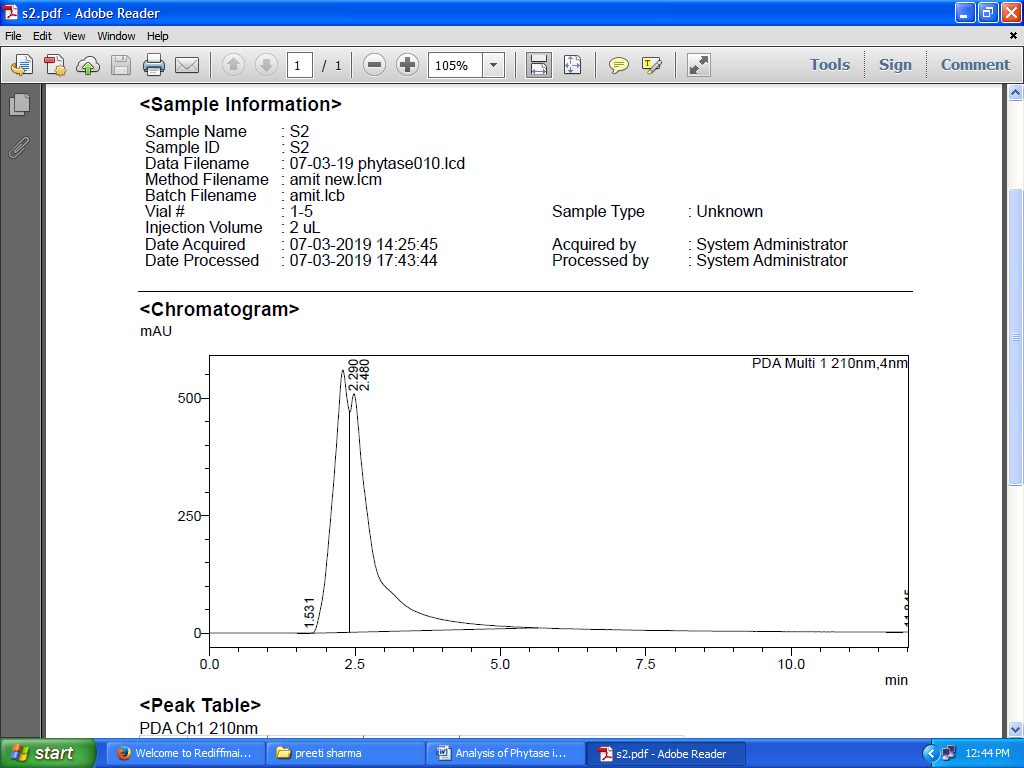


(B)

**Supplementary Figure 2.** HPLC analysis (A) HPLC profile of standard phytase (B) HPLC profile of purified phytase from *P. acidilactici* SMVDUDB2

(A)

(B)

**Supplementary Figure 3.** (A) Effect of pH and (B) temperature on phytase activity of *P. acidilactici* SMVDUDB2. Data are mean ± SE (n = 3).

**Supplementary Figure 4.** Lineweaver-Burk plot of phytase from *P. acidilactici* SMVDUDB2 using sodium phytate as substrate.
